# Classification of common variable immunodeficiency through immunological and clinical phenotyping in Moroccan patients

**DOI:** 10.5339/qmj.2023.sqac.23

**Published:** 2023-11-26

**Authors:** Khaoula Mokhantar, Abir Allaoui, Fatima Ailal, Jalila El Bakkouri, Kaoutar Ouazahrou, Abderrahmane Errami, Ahmed Aziz Bousfiha, Mina Moudatir

**Affiliations:** ^1^Laboratory of Clinical Immunology, Inflammation, and Allergy (LICIA), Faculty of Medicine and Pharmacy, Hassan II University, Casablanca, Morocco Email: khaoulamokhantar@gmail.com; ^2^Department of Internal Medicine, Cheikh Khalifa International University Hospital, Mohammed VI University of Health Sciences, Casablanca, Morocco; ^3^Department of pediatric infectious and immunological diseases, Abderrahim El Harouchi Children Hospital, University Hospital Center Ibn Rochd, Casablanca, Morocco; ^4^Immunology Laboratory, IBN Rochd University Hospital, Casablanca, Morocco; ^5^Department of Internal Medicine, Ibn Rochd UHC, Hassan II University of Casablanca, Casablanca, Morocco

**Keywords:** B-cell subsets, common variable immunodeficiency, classification, flow cytometry, hypogammaglobulinemia

## Abstract

**Objective:** Common variable immunodeficiency (CVID) is a complex inborn error of humoral immunity with complications of both infectious and non-infectious origins. Classifications of CVID patients provide a clearer understanding of the pathogenesis, prediction, and management of non-infectious complications. This study aims to classify Moroccan CVID patients based on the European classification (EUROclass).

**Materials and Methods:** We recruited 20 CVID patients meeting standard diagnostic criteria (5-6). After collecting clinical and demographic data, we used flow cytometry to analyze B-cell subsets and group patients and assess the relation of each group with clinical manifestations.

**Results:** 90% of the patients in our cohort study had a history of respiratory infections. The noninfectious manifestations included splenomegaly, autoimmunity, lymphadenopathy, and granulomatous diseases diagnosed in 50%, 45%, 40%, and 25% of patients, respectively. We observed significant co-occurrence of splenomegaly with autoimmunity and granulomatous diseases to a lesser extent. Patients had a significant reduction in total, switched memory, marginal zone-like, plasma blasts, and a substantial increase in the percentage of activated B cells, suggesting a defect in the late phases of B-cell differentiation. This condition was linked with an increased occurrence of splenomegaly and granulomatous affections. Besides, patients also had an expansion of CD21low B-cells, which was strongly associated with splenomegaly.

**Conclusion:** The classification of the first Moroccan cohort of CVID patients showed agreement with previous results. It suggests the possibility of adopting this approach on a global scale for better diagnosis and follow-up of CVID patients.
